# Obesity among health service providers in Nigeria: danger to long term health worker retention?

**DOI:** 10.11604/pamj.2015.22.1.5586

**Published:** 2015-09-01

**Authors:** Sandra Omozehio Iwuala, Olayinka Olufunmi Ayankogbe, Foluke Adenike Olatona, Michael Adeyemi Olamoyegun, Ukandu OkparaIgwe, Anas Ahmad Sabir, Olufemi Adetola Fasanmade

**Affiliations:** 1 Department of Medicine, College of Medicine, University of Lagos, Idi-Araba, Lagos, Nigeria; 2 Department of Community Health and Primary Care, College of Medicine, University of Lagos, Nigeria; 3 Department of Medicine, LAUTECH Teaching Hospital, and College of Health Sciences, LadokeAkintola University of Technology, Ogbomoso, Oyo State, Nigeria; 4 Lagos University Teaching Hospital, Idi-Araba, Lagos, Nigeria; 5 Department of Medicine, UsmanuDanfodiyo University, Sokoto, Nigeria

**Keywords:** Obesity, health service providers, Nigeria

## Abstract

**Introduction:**

Obesity is a global epidemic. There are rising rates of obesity and its associated disorders, especially in developing countries, including among Health Service Providers (HSPs). Obesity is associated with early retirement, increased morbidity and mortality. Thus, obesity has the potential of reducing long-term retention of HSPs in inadequately staffed health systems of developing countries. This study aimed to determine the magnitude of and factors associated with obesity among HSPs of a tertiary health care facility in Lagos, Nigeria.

**Methods:**

A cross sectional study was carried out with a questionnaire, which included the International Physical Activity Questionnaire short form (IPAQ-SF). Obesity was defined as BMI ≥30kg/m2. Statistical significance was set at p < 0.05.

**Results:**

300 HSPs were recruited, of which 47.7% were medical doctors and dentists, 43.3% were nurses and other categories of HSPs. The mean age and BMI of the HSPs were 39.3(9.0) years and 27.7(4.6) kg/m2 respectively. Eight two (27.3%) HSPs were obese and 134 (44.7%) were overweight, 149(49.7%) had central obesity. After adjusting for confounding variables using multivariate logistic regression, age > 40 years (OR 3.51, p=0.003), female gender (OR 2.84, p=0.007) and earning a monthly salary of ≤ 200,000 naira relative to 201,000-400,000 naira (OR 2.58, p=0.006) were significantly associated with obesity.

**Conclusion:**

Obesity was prevalent among these Nigerian HSPs. This calls for concern, especially with the implication of loosing health workers to obesity related disorders and early retirement.

## Introduction

Obesity, defined as a body mass index of ≥30kg/m2 [1] is a global public health problem, as one in 10 adults are obese [2]. It is associated with a myriad of disorders such as cardiovascular disease, diabetes, hypertension, stroke, sleep apnoea, osteoarthritis, depression, reduced quality of life and several cancers [3–5]. Obesity is linked with huge economic costs [6]. In the United States of America, the cost of obesity in 2008 was 147 billion dollars [6]. During the last 30 years, the prevalence of obesity has increased worldwide and now overweight persons outnumber those with under-nutrition [7, 8]. The developing world, still battling with communicable diseases, is not left out of this global scourge. This is the result of the nutrition transition, reduced physical activity and economic development [9]. Furthermore, there are reports of a more rapid rise in the prevalence of obesity in developing countries compared to developed countries [10]. In some cultures, obesity is admired and seen as a symbol of beauty and virility. Some cultural practices such as “fattening”ceremonies have been reported in various parts of the world such as among the Massa men of Northern Cameroon and Chad, in Tahiti, Nauru, Japanese Sumo wrestlers and the Annang tribe of Calabar, Nigeria [11–17]. Obesity also has implications in the workplace. It is associated with weight discrimination, increased rates of absenteeism, presenteeism, occupational injury, short-term disability and reduced productivity [18–21]. Obese employees have been reported to have the most short-term disability days, costs and least productivity compared with those with lower BMI categories [22]. The work environment has also been shown to contribute to the obesity epidemic. Such “obseogenic”work environment includes shift work, job stress and long work hours [23, 24]. Health service providers (HSPS) such as doctors, nurses, and pharmacists are one of the most important group of workers facing such “obseogenic” work setting. There are conflicting reports about the prevalence of obesity among HSPs who are perceived as role models of healthy behavior in the society. Some studies found obesity prevalence to be lower among health service providers compared to the general population [25–28]. In the USA for example, among 41 professional groups studied, persons of the health diagnosing profession had the lowest obesity rates, being 6.2% in men and 4.3% in women. Conversely, others studies, especially from developing countries have reported higher/similar rates of obesity among HSPs compared with the general population [29, 30]. Furthermore, “obesity approval” and poor perception of weight status among HSPs in Africa has been described [30, 31]. A study among medical health workers in South Africa found that although 73.5% were overweight/obese, 56% were satisfied with their weight [30]. According to the 2010 WHO global infobase, obesity prevalence in Nigeria was 11.0% and 4.3% in Nigerian females and males respectively. A 20% increase of obesity from 2002 to 2010 among adult Nigerians was also described in the same report. Studies on the prevalence of obesity among health service providers in Nigeria is limited. They have been mostly done among health workers [32, 33] (which include administrative staff) or a single professional group such as doctors [29, 34] or nurses [11]. These studies have reported high rates of obesity. among nurses in AkwaIbom state of Nigeria, 62.6% were obese whereas the combined prevalence of overweight and obesity among women 15-49 resident in the same state was (34.8%), according to the 2008 Nigerian Demographic Health Survey Data [11]. Prospective studies have reported increased risk of obesity related NCDs among obese health workers [35]. Obese workers have also been shown to retire earlier than non-obese workers [36], as its presence can create functional disabilities or cause other health problems. Thus obesity is a source of concern for long term health worker retention, especially in developing health systems where the health workforce is inadequate. This study was thus carried out to determine the magnitude of overweight and obesity among health service providers of a tertiary hospital in Lagos, Nigeria and to determine the factors associated with obesity in the HSPs, so as to determine the potential impact of obesity on long term health worker retention.

## Methods

**Study location and design**: a cross sectional, facility-based quantitative study was conducted at the Lagos University Teaching Hospital, a 761 bed and the largest of 5 tertiary health care facilities in Lagos State, the commercial nerve centre in Nigeria. It offers various services such as research, teaching, consultation and clinical services. Health service providers account for 1629 of the 2531 hospital staff.

**Sample size calculation**: the sample size was calculated using the statistical formula for sample size using the Kish and Leslie formula [37], and using a prevalence of overweight and obesity of 73.5% among medical health workers in South Africa [30]. Appropriate adjustment was made using the appropriate formula [38] as the HSPs in the hospital were less than 10,000. The minimum calculated sample size was 253.

**Study population and sampling methodology**: multistage sampling was used to select the 300 HSPs in the hospital. The first stage was the stratification of HSPs into their professions. Then proportionate sampling was used to determine the number of HSPs to be selected from each professional category. Respondents from the professional categories were randomly recruited until the desired sample size for that category was achieved. The HSPs who were contact staff, acutely ill or pregnant were excluded from the study Ethical Approval for the study was obtained from the Health Research and Ethics Committee of the Lagos University Teaching Hospital. Written informed consent was obtained from the study participants.

**Data collection**: the information was collected using a self-administered questionnaire, adapted from other studies encountered during the literature review and from the International physical Activity Questionnaire- short form (IPAQ-SF). Anthropometric measurements (height, weight, waist and hip circumferences) were obtained. The BMI was appropriately derived as the ratio of the weight to the square of the height. Blood pressure was measured according to standard guidelines. Data was collected by the principalinvestigator, and three trained research assistants, between July and August 2013.

**Outcome and independent variables**: obesity defined as a BMI ≥30kg/m2 was the main outcome variable in this study [39]. Body mass Index was categorized according to the World Health organization weight criteria and central obesity was defined as a waist circumference of >88 cm in females >102 cm in males [39]. The independent variables were age, sex, marital status, ethnicity, average monthly salary, number of years of experience, physical activity levels, blood pressure and professional category. Physical activity levels were categorized into two: meeting the recommended levels of PA and not meeting the recommended levels of PA. Persons with high and moderate levels of PA according to IPAQ scoring guidelines met the recommended levels of PA while those with low levels of PA did not meet the recommended levels of PA [40]. There were 3 professional categories. Medical doctors and dentists were categorized as “doctors”, nurses as “nurses” and all other HSPs which included 12 pharmacists, 2 physiotherapists, 7 laboratory scientists, 1 Optometrist, 1dietician, 1 Pharmacy technician, 1 Radiographer and 1 Social worker as “others”.

**Data management and statistical analysis**: microsoft excel was used for cleaning the data while statistical analysis was done using the statistical package for social sciences, SPSS version 20.0 (IBM SPSS Inc. Chicago Illinois). Continuous variables were expressed as means and standard deviation or median and interquartile range. Categorical variables were expressed as frequencies with accompanying percentages. Differences between groups were compared using the chi-square and fisher's exact test for categorical variables. Odds ratio and the corresponding 95% confidence intervals (CI) were presented. The student t test was used to compare difference between groups for continuous variables. Comparison of variables that were not normally distributed was done using non-parametric statistical tests. Multi-variate logistic regression analysis was used to determine the factors associated with obesity. The factors that were significantly associated with obesity (p < 0.05) on univariate analysis were put into a model to adjust for confounding factors. Statistical significance was set at p value of < 0.05.

## Results

**Sociodemographic and work related characteristics of the study population**: Table 1 shows the characteristics of the study population. The mean age of the study population was 39.3 (9.0) years. There were 199 (66.3%) females and 101 (33.7%) males. The mean BMI of the HSPs was in the overweight category 27.7 (4.6) kg/m2 and physical inactivity was prevalent (79.2%) among the HSPs.(2 persons were voided from the analysis for PA according to IPAQ scoring guidelines) [10].

**Table 1:** Characteristics of the Health Service Providers

Characteristic	Frequency N=300	Percent (%)
**Age group (years)**		
≤40	185	61.7
>40	115	38.3
**Sex**		
Female	199	66.3
Male	101	33.7
**Marital status**		
Single	57	19.0
Married/Separated/Widowed	243	81.0
**Ethnic group**		
Yoruba	172	57.3
Igbo	83	27.7
Others	45	15.0
**Average monthly salary (naira)**		
≤ 200,000	149	49.7
201,000-400,000	125	41.7
>400,000	26	8.7
**Number of years of experience**		
< 5	43	14.3
5-10	103	34.3
>10	154	51.3
Median (IQR)	11(14)	
**Profession**		
Doctors/Dentists	143	47.7
Nurses	130	43.3
Others	27	9.0
**Body Mass Index (kg/m** ^**2**^ **)**	27.7	4.6
**Waist circumference (female)**	119.6	10.9
**Waist circumference (male)**	84.1	9.2
Physical inactivity*	236/298	79.2
**Systolic Blood pressure (mmHg)**	121.1 (17.5)	
**Diastolic Blood Pressure (mmHg)**	76.5 (11.9)	

Values are mean (SD) or frequencies with accompanying percentages

* Two persons were voided from the analysis according to the IPAQ scoring guidelines

**Obesity among the HSPs**: eighty-two (27.3%) HSPs were obese while 134(44.7%) HSPs were overweight (i.e. BMI ≥25kg/m2). Central obesity was present among 149(49.7%) HSPs, comprised of 13(12.9%) males and 136 (68.3%) females.

**Association of obesity with other cardiovascular risk factors**: the association of obesity with other cardiovascular risk factors is shown in Table 2. Obese HSPs had a higher mean WC, systolic and diastolic blood pressures and physical inactivity levels (p <0.05).

**Table 2:** Association of obesity with other cardiovascular risk factors

Characteristic	Obese(N=82)	Non-obese (N=218)	P value
Systolic Blood pressure (mmHg)	126.3 (19.5)	119.1 (16.3)	0.001
Diastolic Blood pressure (mmHg)	81.9 (12.1)	74.5(11.3)	<0.001
Waist circumference _female_ (cm)	101.9 (8.3)	87.2 (8.6)	<0.001
Waist circumference _male_ (cm)	103.8 (8.5)	89.1 (7.2)	<0.001
**Physical inactivity**			
Yes	67(28.4)	169 (71.6)	0.451
No	14(22.6)	48(77.4)	

**Factors associated with obesity among the HSPs**: univariate analysis showed that obesity was associated with age > 40 years, (p < 0.001), gender (p=0.001), marital status (p=0.030), average monthly salary (p=0.042), no of years of experience (p=0.001), and profession (p=0.041) (Table 3). These variables that were significantly associated with obesity (p < 0.05) were put into a multivariate logistic regression model. Age > 40 years (OR 3.51, p=0.003), male gender (OR=2.84, p=0.007) and earning a monthly salary of ≤200,000 naira relative to 201,000-400,000 naira (OR 2.58, p=0.006) were the factors that remained significant on the multivariate logistic regression analysis as shown in Table 4.

**Table 3:** Association of obesity with socio-demographic and work related characteristics

Characteristic	Obese (N=82)	Non-obese (N=218)	P value
**Age group (years)**			
≤ 40	34(18.4)	151(81.6)	<0.001
> 40	48(41.7)	67(58.3)	
**Mean age (SD)**	43.0(8.8)	37.9(8.7)	<0.001
**Sex**			
Female	66(33.2)	133(66.8)	0.001
Male	16(15.8)	85(84.2)	
**Marital status**			
Single (never married)	9(15.8)	48(84.2)	0.030
Married/ Separated/ Widowed	73(30.0)	170(70.0)	
**Ethnic group**			
Yoruba	44(25.6)	128(74.4)	0.581
Igbo	23(27.7)	60(72.3)	
Others	15(33.3)	30(66.7)	
**Average monthly salary (naira)**			
≤ 200,000	47(31.5)	102(68.5)	0.042
201,000-400,000	25(20.0)	100(80.0)	
>400,000	10(38.5)	16(61.5)	
**Number of years of experience**			
< 5	6(14.0)	37(86.0)	0.001
5-10	20(19.4)	83(80.6)	
>10	56(36.4)	98(63.6)	
**Profession**			
Doctors	30(21.0)	113(79.0)	0.041
Nurses	45(34.6)	85(65.4)	
Others	7(25.9)	20(74.1)	

**Table 4:** Multivariate logistic regression of the factors associated with obesity

Characteristic	Odds ratio	95% CI	P value
**Age group (years)**			
≤ 40 (reference)	-		
> 40	3.51	1.54-8.01	0.003*
**Sex**			
Male (reference)	2.84	1.33-6.05	0.007*
Female	-		
**Marital status**			
Single (never married)	1.63	0.69-3.85	0.264
Married/ Separated/ Widowed (reference)	-		
**Average monthly salary (naira)**			
≤ 200,000	2.58	1.32-5.07	0.006*
201,000-400,000 (reference)	-		
>400,000	1.92	0.64-5.75	0.246
**Number of years of experience**			
< 5 (reference)	-		
5-10	1.73	0.61-4.91	0.303
>10	1.76	0.52-6.04	0.367
**Profession**			
Doctors (reference)	-		
Nurses	0.53	0.21-1.32	0.177
Others	0.66	0.21-2.10	0.485

* Statistically significant

## Discussion

The aim of this study was to determine the rate and factors of overweight and obesity among HSPs in a tertiary health care facility in Nigeria. In this study, over a quarter (27.3%) of the HSPs were obese and almost three quarters (72.0%) were either overweight/obese. Central obesity, which is more strongly associated with cardiovascular disease was even more frequent (49.7%) compared to global obesity among these HSPs Obese HSPs had higher mean blood pressure compared to those who were not obese. The rate of obesity among the HSPs in this study was higher than most reports of obesity among Nigerian populations [41–46] and workers [47–49]. A 2008 WHO estimate of obesity in Nigerians was 6.5%, while a systematic review that included 4 population based studies of urban and semi-urban communities reported obesity rates ranging from of 8.1% to 22.2% in Nigerian populations. Although, one of the 4 studies included in the systematic review was done in Lagos, the same location as the study site albeit in the community, the prevalence of obesity was 22.2%, [50] lower than the found among HSPs in this study. The difference could be due to the fact that these studies were community based, whereas this study was done among HSP who have a higher average income compared to the general population. Higher income has been associated with higher prevalence of obesity in Nigerian studies [51, 52]. Reports of higher [53] or similar [54] obesity rates among HSPs compared to the general population have been reported in Africa where obesity is often admired and perceived as a sign of affluence and good health [31]. In a study of 100 black medical health care workers in South Africa, the reported prevalence of overweight/obesity was 60.5% and 76.5% in the male and female (HCW) respectively when the prevalence of overweight/obesity was 49% in black men and 75% of black women in the general population [53]. Another study on obesity among female nurses in AkwaIbom state of Nigeria published in 2009, found 62.6% of the 500 nurses to be obese [11]. The prevalence of obesity in among these nurses was much higher than the combined overweight and obesity rates (34.8%) in women aged 15-49 resident in the same state, according to data from the 2008 Nigerian Demographic and Health Survey (NDHS) [52].

In contrast to the findings from this study, most reports from developed countries suggest lower obesity prevalence among health service providers compared to the general population and other categories of workers. [25, 27, 55]. In a study on health practices of Canadian physicians, only 8% were obese [27]. There was also lower rate of overweight persons among the physicians compared to the general population [27]. among 41 professions studied in the National Health Interview Survey of the United States of America (1986 to 2002), persons of the health diagnosing professions had the lowest obesity rates (being 6.2% in men and 4.3% in women.) while the highest obesity rates (31.7%) were documented among motor vehicle operators [25]. In this study, the determinants of obesity among the HSPs after adjusting for confounding variables in the multivariate logistic regression model were older age, male gender and lower income. Other studies done among HSPs have shown older age to be associated with obesity as found in this study [56–58]. Advancing age results in slowing of the metabolic processes, reduced physical activity and a decrease in the proportion of skeletal muscle mass. The association of female gender with obesity among HSPs has been reported by other workers [58–60]. In this study, persons with lower monthly income of ≤200, 000 naira (approximately 1200 dollars) monthly were almost 3 times more likely to be obese compared with persons in the 200,000-400,000 naira category. This is contrary to findings from the study among chief executives in Jos, Nigeria in which persons who earned <100,000 naira monthly were more likely to be obese compared to those who earned >100,000 naira [51]. Similarly, the associations of higher income [61] and higher socioeconomic status [45, 62] with obesity in population based studies in Nigeria has been reported.

The difference in the findings of the association between income and obesity can be explained by the relative affluence of HSPs. In developed countries, lower income is associated with higher obesity rates lower income being associated with obesity can be explained by the relatively high socioeconomic status of HSPs compared with the general population. Studies from more affluent societies have reported the association of lower income with higher obesity rates, as found in this study. These HSPs are a relatively affluent group of Nigerians, this could the similarity in association of lower income with obesity observed in more affluent societies. The findings from this study of a high rate of obesity including central obesity, as well as low levels of PA among HSPs have several implications. In recent years, the study of the health workforce has gained prominence, because the link between human resources and health system effectiveness has become clearer [63]. High rates of obesity in the health workforce, which is still grossly inadequate in developing countries, will translate to reduced productivity, contributed to by absenteeism and work related injuries. Furthermore, the burden of obesity associated NCDs such as diabetes, hypertension, cardiovascular disorders, all of which increase morbidity and mortality among these HSPs is likely to be high. This can lead to problems with long term retention of health workers who are already in short supply in developing countries, as obesity has been shown to be a factor in early retirement [36]. This association of obesity with NCDs among Nigerian HSPs is a thrust for further research. Lastly, if HSPs are to be agents of change in this obesity pandemic, by being good role models, as well as health promotors and educators, they need who start practicing what they preach. The perception of Nigerian HSPs towards obesity and as role models of healthy weight needs further exploration. It is recommended that HSPs should empowered to be good role models in the obesity pandemic though collaborative efforts by nutrition and physical therapy departments. As part of workplace health promotion programmes. The limitations of this study include the cross sectional study design as well as its setting in an urban, tertiary health care facility. Hence, the results may not be generalized to HSPs in rural or semi urban locations. However, anthropometric measurements were made using standard methods and a questionnaire (IPAQ-SF) which has which used in diverse cultural contexts including in Nigeria [44] was used to assess physical activity.

## Conclusion

The high burden of overweight and obesity among HSPs in this study calls for concern and action. Health service providers in Nigeria need to be sensitized and empowered to be roles models of healthy weight in the society. Workplace health promotion programs focusing on availability of healthy food choices and opportunities for increased physical activity should be established to encourage long term health worker retention.
